# Unraveling Cajal's view of the olfactory system

**DOI:** 10.3389/fnana.2014.00055

**Published:** 2014-07-02

**Authors:** María Figueres-Oñate, Yolanda Gutiérrez, Laura López-Mascaraque

**Affiliations:** Department of Molecular, Cellular, and Developmental Neurobiology, Instituto Cajal (CSIC)Madrid, Spain

**Keywords:** olfactory bulb, olfactory cortex, olfactory epithelium, glia, neuron

## Abstract

The olfactory system has a highly regular organization of interconnected synaptic circuits from the periphery. It is therefore an excellent model for understanding general principles about how the brain processes information. Cajal revealed the basic cell types and their interconnections at the end of the XIX century. Since his original descriptions, the observation and analysis of the olfactory system and its components represents a major topic in neuroscience studies, providing important insights into the neural mechanisms. In this review, we will highlight the importance of Cajal contributions and his legacy to the actual knowledge of the olfactory system.

## Introduction

Santiago Ramón y Cajal is called the father of modern neuroscience for our current understanding of the nervous system really began through his work. Cajal postulated the main principle of neuroscience, the Neuron Doctrine, which recognizes the neuron as the basic anatomical and functional unit of the nervous system (Ramón y Cajal, 1891). His view was opposed to the reticular theory developed by Golgi (1873). The principal advantage that Cajal had over his contemporaries was the better understanding of the Golgi method allowing thus its correct interpretation. With this edge Cajal was able to give a successful explanation to the static view of the sections impregnated by the Golgi method. He was even able to make predictions on physiological brain properties that are being demonstrated nowadays thanks to more sophisticated techniques. Based on observations done using the Golgi method Cajal concluded: “It happens sometimes that the reaction of Golgi runs from one fiber to another when two of them intersect, resembling branches or anastomotic examples. This error can only be avoided by using high magnifying lenses and not giving credit to other branches other than those that appear in the focal plane and on the level of those triangular thickenings that are never absent in cases of a legitimate branch” (Ramón y Cajal, 1890b). In birds' cerebellum, Cajal correctly described that the surface of Purkinje cells “appears bristling with thorns or short spines” (Ramón y Cajal, 1888); Golgi instead, rejected the existence of these spines, considering them artifacts of the silver staining technique. He also established the connections between neurons and drew the maps of the trajectory of nerve currents and impulses that led him to formulate the Law of Dynamic Polarization: “The protoplasmic expansions, dendrites, and the cellular body have axipetal conduction (i.e., toward the axon); whereas the axon has dendrifugal and somatofugal conduction (i.e., it comes from the dendrites or the cellular body)” (Ramón y Cajal, 1899). Furthermore, Cajal described the growth cone as a “concentration of protoplasm of conical form, endowed with amoeboid movements” (Ramón y Cajal, 1890a). Another of Cajal's contributions was the formulation of the Neurotropic Theory (Ramón y Cajal, 1892a); which shows how nerve cells find their way to their targets during development. In the formulation of these Laws explaining the morphological and functional organization of the nervous system, the analyses of the olfactory system was critical; this due to its accessibility, its orderly organization in layers and the easy identification of the main direction of the nervous message flow. Thus, the aim of this article is to give a brief outline of Cajal's main contributions to the knowledge of the olfactory system along with some key developments in our current understanding of this system.

## Olfactory circuit

“The flow of the nervous movement in the bulb would be the following: the olfactory imprint is collected in the mucosa by the peripheral expansion of the bipolar cells and is then transferred to the glomeruli where both the mitral corpuscles as well as the pyramidal or fusiform cells from the molecular layer collect said imprint to raise it to the brain. […] In summary, there are two main junctions: one in the glomeruli and another one in the cortex of the olfactory lobe. In each one of these junctions the movement acquires more diffusion, partaking in its conduction an increasingly larger number of nervous corpuscles” (Ramón y Cajal, 1892b).

**Figure 1:**
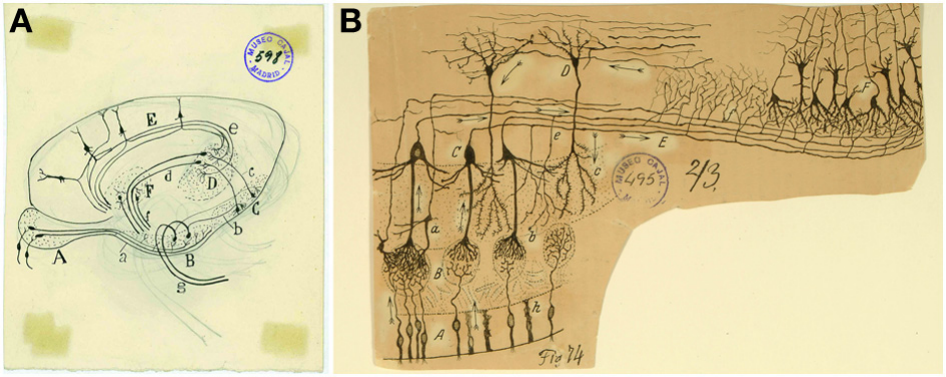
**Overall view of the olfactory circuit. (A)** Unpublished original Cajal cartoon of the olfactory system. **(B)** Cajal's diagram outlining the circuitry and the trajectory of the nerve impulse (arrows). *(A)* Bipolar cells of the olfactory mucosa. *(B)* Olfactory glomeruli. *(C)* Mitral cells. *(D)* Grains. *(E)* External root (lateral olfactory tract). *(F)* Olfactory cortex. *(a)* Small tufted cell; *(b)* main dendrite of a mitral cell; *(c)* terminal branch of a grain; *(e)* recurrent collaterals of a mitral cell; *(g)* surface triangular cells of the olfactory cortex; *(h)* epithelial cells of the nasal mucosa (Ramón y Cajal, 1894). Cajal Legacy (Instituto Cajal, CSIC, Madrid, Spain).

The olfactory system represents an excellent model of the cellular interaction between the periphery and the central nervous system. In the nasal cavity is located the olfactory epithelium (OE) where the olfactory sensory neurons (OSNs), in direct contact with the environment, are contained. OSNs project their axons, through the cribriform plate, to contact target cells in the olfactory bulb (OB). OB projection cells send the olfactory signal to the olfactory cortex (OC), which includes the olfactory tubercle, piriform cortex, amygdala, and entorhinal cortex. The olfactory information is then further transmitted to the thalamus, hypothalamus, or hippocampus (Figure 1A). One of Cajal's most important contributions, the *Law of dynamic polarization*, was possible by the observation of the direction of the signal flow from one neuron to the next in this system. In particular, he used arrows to represent in his histological drawings the flow of information from the periphery (OE) to the OB in the brain and then onto the OC (Figure 1B): “Excitation is conducted at the glomeruli, where numerous olfactory fibers end. Here, the motion is transmitted along several currents directed along the path of the projection cells (mitral or superior, medial, and inferior tufted cells), from the intraglomerular tufts, to the axis-cylinders and their cerebral endpoints in the olfactory centers” (Ramón y Cajal, 1890b). Thus, even without a functional frame, Cajal proposed the direction of the information flow that was later corroborated by physiological studies (reviewed in Shepherd and Erulkar, 1997), although the presence of axonless granule cells in this system challenged the *Law of dynamic polarization* (Shepherd et al., 2007; Sassoè-Pognetto, 2011).

**Figure 2:**
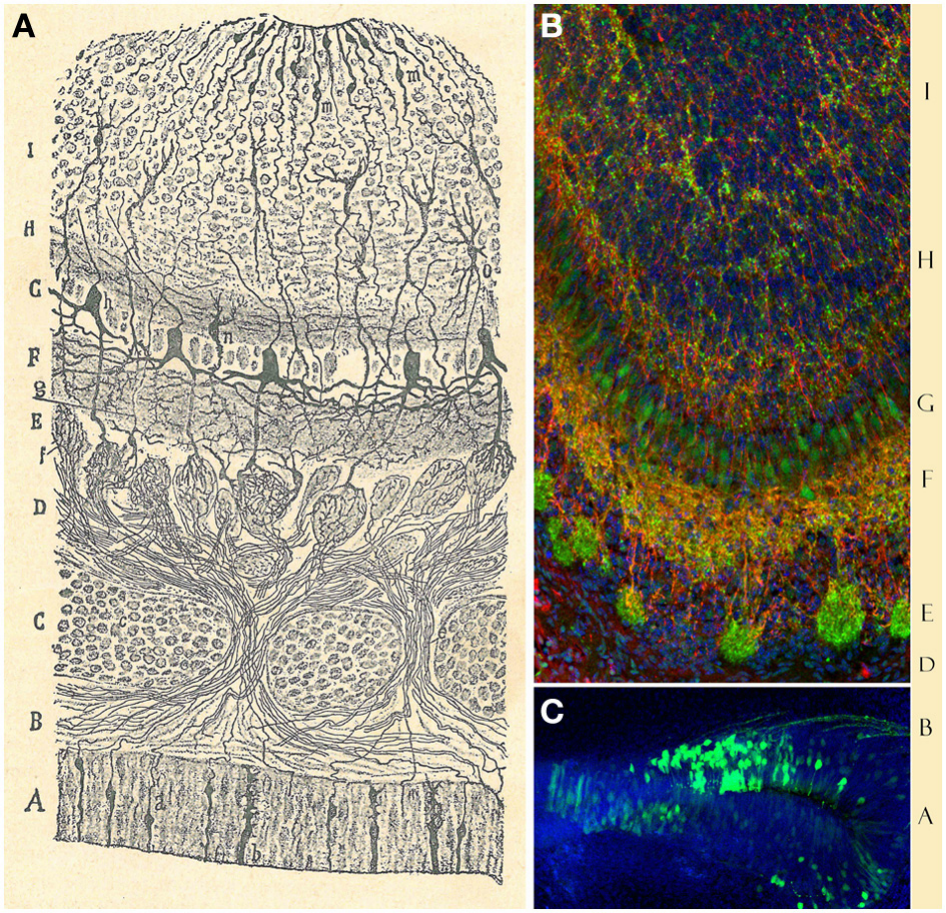
**Olfactory bulb and epithelium. (A)** Original Cajal drawing showing the “antero-posterior view of the olfactory bulb and nasal mucosa of the newly-born mouse.” *(A)* olfactory epithelium; *(a)* olfactory sensory neuron; *(b)* sustentacular cell. *(B)* Dermis. *(C)* Ethmoid bone. *(D)* Fibrillar layer. *(E)* Glomerular layer. *(F)* Inferior molecular layer. *(G)* Mitral cells layer. *(H)* Superior molecular region. *(I)* Granular zone. *(c)* Cartilage. *(e)* Olfactory nerve. *(f)* Arborization of the olfactory fibers within the glomeruli. *(g)* Central cyllinder-axis of an inferior tufted cell. *(h)* Mitral cell. *(i)* Grains. *(j)* Epithelial cells. *(n)* Lower grain. *(o)* Large stellated cell (Ramón y Cajal, 1890b). **(B)** Immunohistochemical staining for Dab1 protein (reelin signaling mediator, green) and Map2a,b protein (microtubule associated protein, red) on mouse olfactory bulb sections at P3. Nuclei counterstained with Hoechst (blue) (Martín-López et al., 2011). **(C)** Olfactory sensory neurons (green) at E17, labeled after *in utero* electroporation of an EGP-expressing plasmid into the olfactory placode at E11. Nuclei counterstained with Hoechst (blue). Sagittal mouse brain section.

Cajal's detailed study of the olfactory system and its components (Figure 2A) (Ramón y Cajal, 1890b) laid the foundations for later contemporary studies (Figures 2B,C). In his book “*Recuerdos de mi vida*” (Ramón y Cajal, 1917), he defines the OB as an accessible and regular structure, comparable to the cerebellum and retina. In this system, once again, he evidenced the nerve propagation by contact and the important role of dendrites: “The history of the physiological interpretation of the structure of the olfactory bulb provides a typical case of the crippling influence of theoretical prejudices. Golgi had already discovered before us the most important facts of that structure, the singularly invaluable concurrency within the glomeruli of the olfactory fibers, on the one hand, and the dendritic tuft of mitral cells on the other; but his rigid conception of the diffuse nervous network did not allow him to recognize the great physiological scope of such provision” (Ramón y Cajal, 1917).

These characteristics are reinforced by the incorporation of new cellular elements not only during development, but also during adulthood (Altman, 1969; Lois and Alvarez-Buylla, 1994). The plastic process in the OB is the result of the combination of cellular contributions from either the telencephalic subventricular zone as a part of the central nervous system, and the olfactory placode/epithelium, which “represents a peripheral nervous center” (Ramón y Cajal, 1892b).

## Olfactory epithelium

“The olfactory mucosa contains the nervous cells from where the olfactory fibers that reach the brain through the ethmoid's lamina cribrose; it thus represents a peripheral nervous center. […] The bipolar or olfactory cell represents the real reception organ of the odorant impulse or stimulus” (Ramón y Cajal, 1892b).

**Figure 3:**
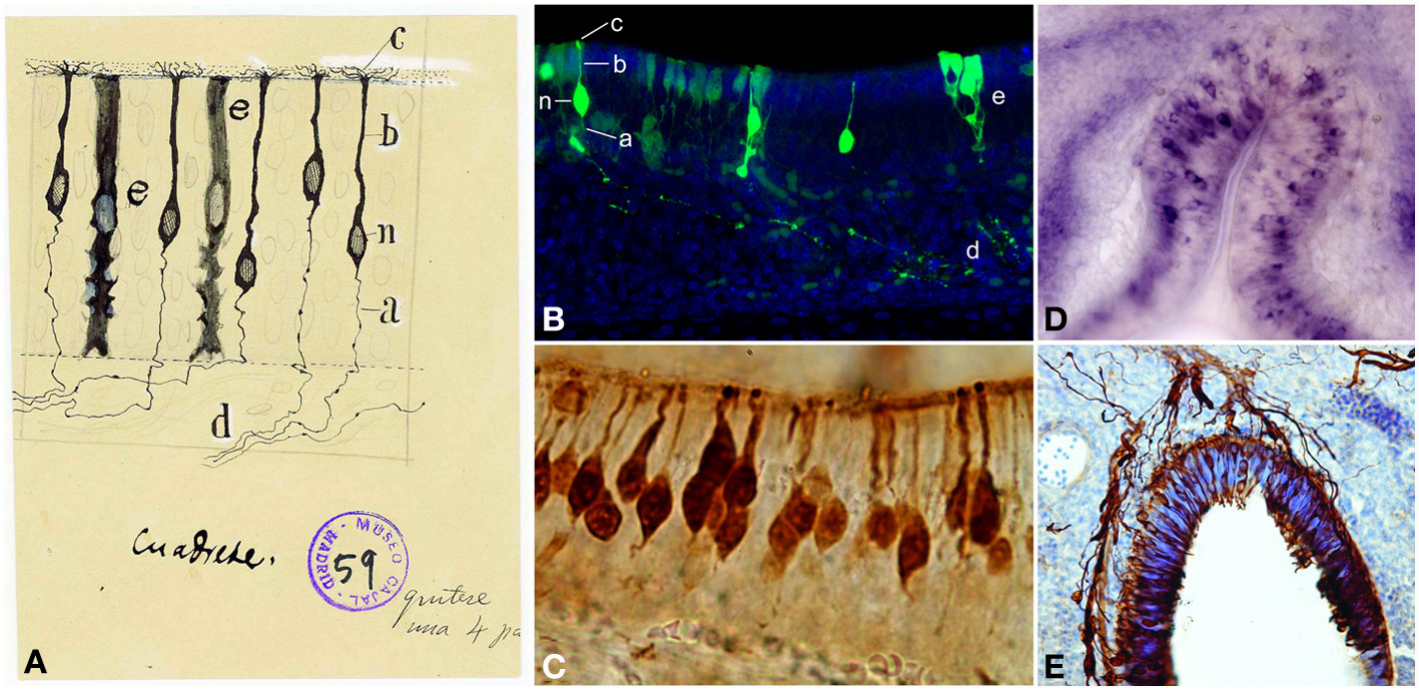
**Olfactory sensory neurons (OSNs). (A)** Cajal drawing illustrating the cell types and their morphologies in the olfactory epithelium. OSN components: *(n)* nucleus; *(b)* dendrite; *(c)* apical cilia; *(a)* axon. *(d)* OSNs axons bundle. *(e)* supporting or sustentacular cell. The drawing shows the handwriting of Cajal with specific instructions for the required reduction publication factor in the margins (Ramón y Cajal, 1917). Cajal Legacy (Instituto Cajal, CSIC, Madrid, Spain). **(B)** Olfactory epithelium labeled E18 cells after *in utero* electroporation of an EGP-expressing plasmid injected into the olfactory placode at E14 (green). Hoechst (blue). Sagittal mouse section. *a–e, n* correspond to the counterpart structures labeled by Cajal in **(A)**. **(C)** Immunohistochemistry for the Tuj1 marker in mouse olfactory epithelium shows both mature and immature OSNs. **(D)**. *In situ* hybridization for *Nrp-II mRNA* in coronal sections of mouse olfactory epithelium at E14. **(E)** Immunohistochemistry for Tuj1 marker in a mouse olfactory placode coronal section at E11. Panels **(C–E)** were taken by Albert Blanchart.

The olfactory epithelium is the place where volatile odorant molecules are initially detected and it is composed by three cell types: OSNs, supporting cells and basal cells. OSNs (Figure 3) are bipolar cells located in the intermediate OE region, distributed between the supporting cells. Their apical processes end at the lumen in non-motile cilia, while the thinner descending axon “gives neuronal character to the bipolar cell” (Ramón y Cajal, 1892b) and transmits the impulse to the OB. The supporting or sustentacular cells exhibit an irregular morphology, but their nuclei are mostly located apically, thereby being narrower on their basal side (Figures 3A,B). The characteristic morphology of these cells offers “numerous facets or hollow molds in order to adapt to the bipolar corpuscles […] and their mission appears to be no other than preventing any contact between them, avoiding any horizontal current communication” (Ramón y Cajal, 1892b). Basal cells, not described by Cajal, form a single cell layer in the basal lamina, near the underlying bone of the OE (Retzius, 1892). They have a constant turnover (Graziadei, 1973) being the precursors of OSNs (Suzuki et al., 2013).

One of the main advances in the study of this system was the cloning of the olfactory signal transduction molecules, in particular the odorant receptors (Buck and Axel, 1991) located on the OSNs cilia. In mice there are over five million OSNs, each expressing just one among the thousand odorant receptor genes (Zhang and Firestein, 2002). These chemosensory receptors are odorant-binding proteins with seven transmembrane domains coupled to G-proteins. Each receptor is codified by the allele of a single gene (Buck and Axel, 1991) and binds only odor molecules of a certain family. They are responsible of transforming the chemical information into electric signals in the olfactory circuit. Genetic tools reported that OSNs, expressing a given odorant receptor, are intermingled and randomly distributed within four large OE zones. These zones are symmetric in both sides of the nasal cavities and are divided based on the expression pattern of some odorant receptors (Ressler et al., 1993; Vassar et al., 1993). Furthermore, OSNs expressing the same receptor converge upon a stereotypical pair of glomeruli (Mombaerts et al., 1996). Nonetheless, the mechanisms by which a set of OSNs, expressing certain odorant receptor, innervates a discrete amount of glomeruli are not well-known; although it seems to be dependent on environmental cues, as well as on intrinsic OSN/odorant receptor factors (reviewed in Mombaerts, 2006; Blanchart and López-Mascaraque, 2011).

**Figure 4:**
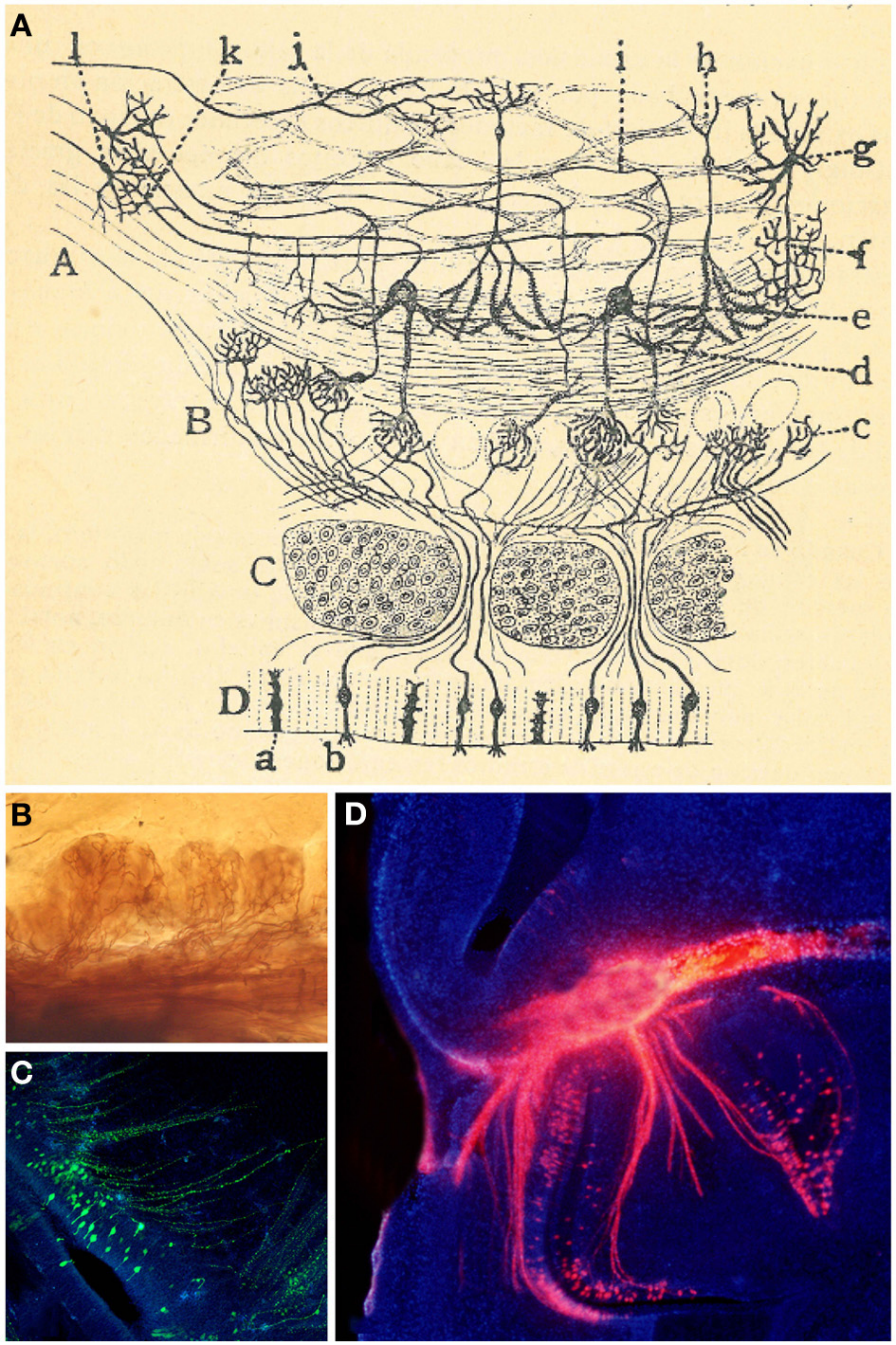
**Connection between the olfactory epithelium and the olfactory bulb. (A)** Cajal schematic drawing of the mammal's olfactory system. *(A)* Olfactory lobe. *(B)* Olfactory bulb's glomerular layer. *(C)* Cribriform plate. *(D)* Olfactory epithelium or nasal mucosa; *(a)* supporting cells; *(b)* OSNs (Ramón y Cajal, 1892b). **(B)** Axonal arborizations of OSNs axons in several glomeruli stained with the Golgi method. **(C)** Olfactory sensory neurons (green) at E17, labeled after *in utero* electroporation of an EGP-expressing plasmid into the olfactory placode at E11. Nuclei counterstained with Hoechst (blue). Sagittal mouse section. **(D)** Retrogradely labeled OSN cells after a DiI crystal application into the OB (red). Hoechst (blue).

Cajal showed that OSNs axons ended into the glomeruli (Figure 4A): “This fibril goes through a part of the dermis indivisible and without anastomosing, then gathering with others in tight bundles, goes upwards later, always preserving its individuality, through the ethmoid's lamina cribrosa and assaults, finally, the olfactory bulb, ending arborizing in the thickness of one glomeruli of this central nervous system organ” (Ramón y Cajal, 1892b). Even when the past decades have seen enormous achievements by the implementation of new technologies, Cajal's morphological descriptions provided the basis for subsequent studies. *In situ* hybridization and immunohistochemistry revealed the molecular features of OSNs (Figures 3C–E). Later, the use of HRP, retrograde fluorescent markers (Fast Blue, Diamidino Yellow), biotinylated dextrans and lipophilic fluorescent tracers (e.g., DiI, DiO, DiA) confirmed the pathway of OSN axons from the periphery to the OB. At this respect, Figure 4D shows the path of retrogradely OSN labeled cells after a DiI injection into the OB. Furthermore, techniques such as *in utero* electroporation of an EGP-expressing plasmid used to study the olfactory pit cell migrations allowed also a further visualization of these nerve bundles (Figure 4C). These axons do not ramify until they reach the glomeruli (Figure 4B), where they will make contacts with the dendrites of the projection neurons. It is within these specialized structures where the information from the periphery is integrated and then conducted to the rest of the brain. Additionally, the development of the OB is not dependent on the presence of the OE or the synaptic input from the OSNs (López-Mascaraque et al., 1996; López-Mascaraque and De Castro, 2002), although OSN axons are critical during OB layering in the final orientation of mitral cells (López-Mascaraque et al., 2005).

## Spatial cell arrangements in the olfactory bulb

Next station in the olfactory pathway is the olfactory bulb: “the olfactory nerves, which bore into the cranium base through several holes in considerable numbers, and assault the olfactory bulb where they end” (Ramón y Cajal, 1890b). One of the most important of Cajal findings was the demonstration of the entire course of the olfactory fibers. Cajal made the real assumption that these fibers come from the mucosa (OE) and end into the glomerulus at the OB not as a network, as Golgi thought, but by free varicose arborizations.

The OB has a well-defined laminar structure and is formed by different cell populations divided into projection neurons (mitral cells and some tufted cells), interneurons (periglomerular cells, external tufted cells, short axon cells, granule cells, Van Gehuchten cells, and Blanes cells) and glial cells (astrocytes, oligodendrocytes, olfactory ensheathing cells, NG2, and microglia). The innermost part of the OB, the ependymal zone, contains progenitor cells.

**Figure 5:**
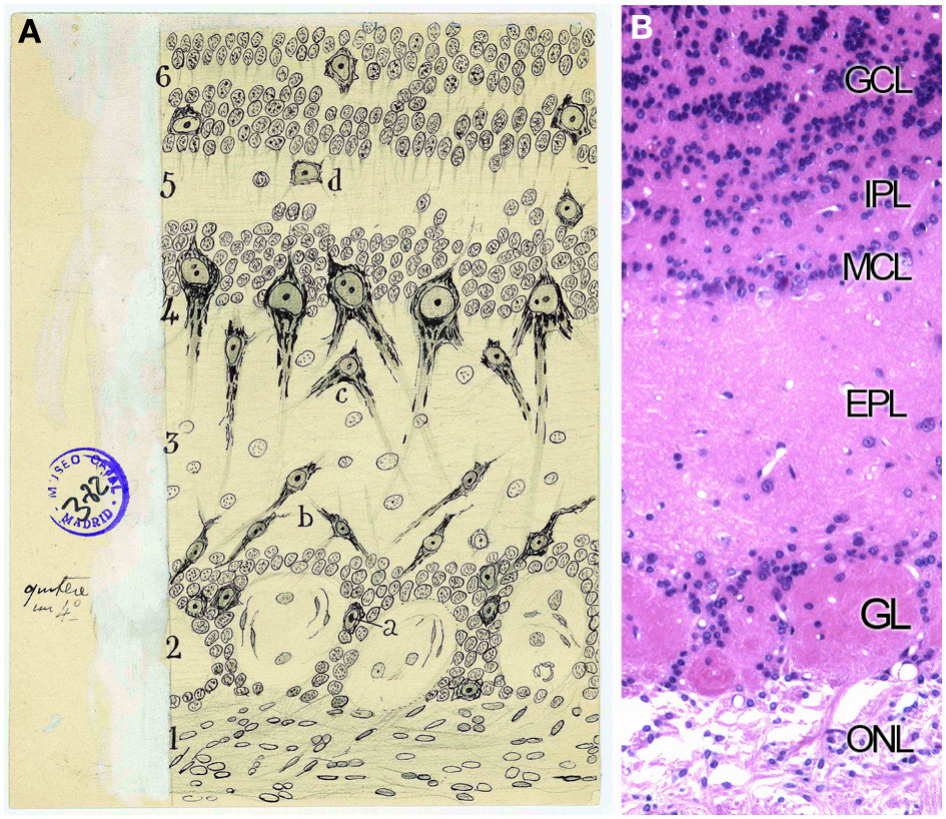
**Layers distribution of the Olfactory bulb. (A)** Original Cajal drawing of the frontal section of the rabbit olfactory bulb (Ramón y Cajal, 1901). *(1)* Nerve layer (ONL). *(2)* Glomerular layer (GL). *(3)* External plexiform layer (EPL). *(4)* Mitral cells layer (MCL). *(5)* Inner plexiform layer (IPL). *(6)* Grains and white matter layer (GcL). *(a)* Peripheral tufted cells; *(b)* middle; *(c)* internal *(d)* short axon cells. Cajal Legacy (Instituto Cajal, CSIC, Madrid, Spain). **(B)** Olfactory bulb mouse coronal section of hematoxylin and eosin nuclei staining showing the different layers described by Cajal in **(A)**.

Golgi considered the OB formed by three layers (Golgi, 1875) while Schwalbe (1881) proposed six layers. The definitive description of cell types and disposition in six layers was given by Cajal and his disciples (Ramón y Cajal, 1890b; Blanes, 1898). From the outside in, the OB is organized in the following layers: the olfactory nerve layer (ONL), glomerular layer (GL), the external plexiform layer (EPL), the mitral cell layer (MCL), the internal plexiform layer (IPL) and the granule cell layer (GcL) (Figures 5A,B). Cajal stated that the ONL was formed by unbranched *“nerve fibrils”* which preserve the same thickness along their trajectory from the OE. Besides, this layer contains an extremely interesting population restricted exclusively to the olfactory system regions, the olfactory ensheathing cells (Valverde and López-Mascaraque, 1991). During development, olfactory ensheathing cells coexist with astrocytes as part of the migratory mass (Doucette, 1990; De Carlos et al., 1996; Blanchart and López-Mascaraque, 2011; Blanchart et al., 2011). Olfactory ensheathing cells maintain certain progenitor characteristics (Schwarting et al., 2007) and are responsible, among other things, for the permissibility within the OB to OSNs axons growth during development and adulthood, thus being a key component of the ability of the OE to continually regenerate.

**Figure 6:**
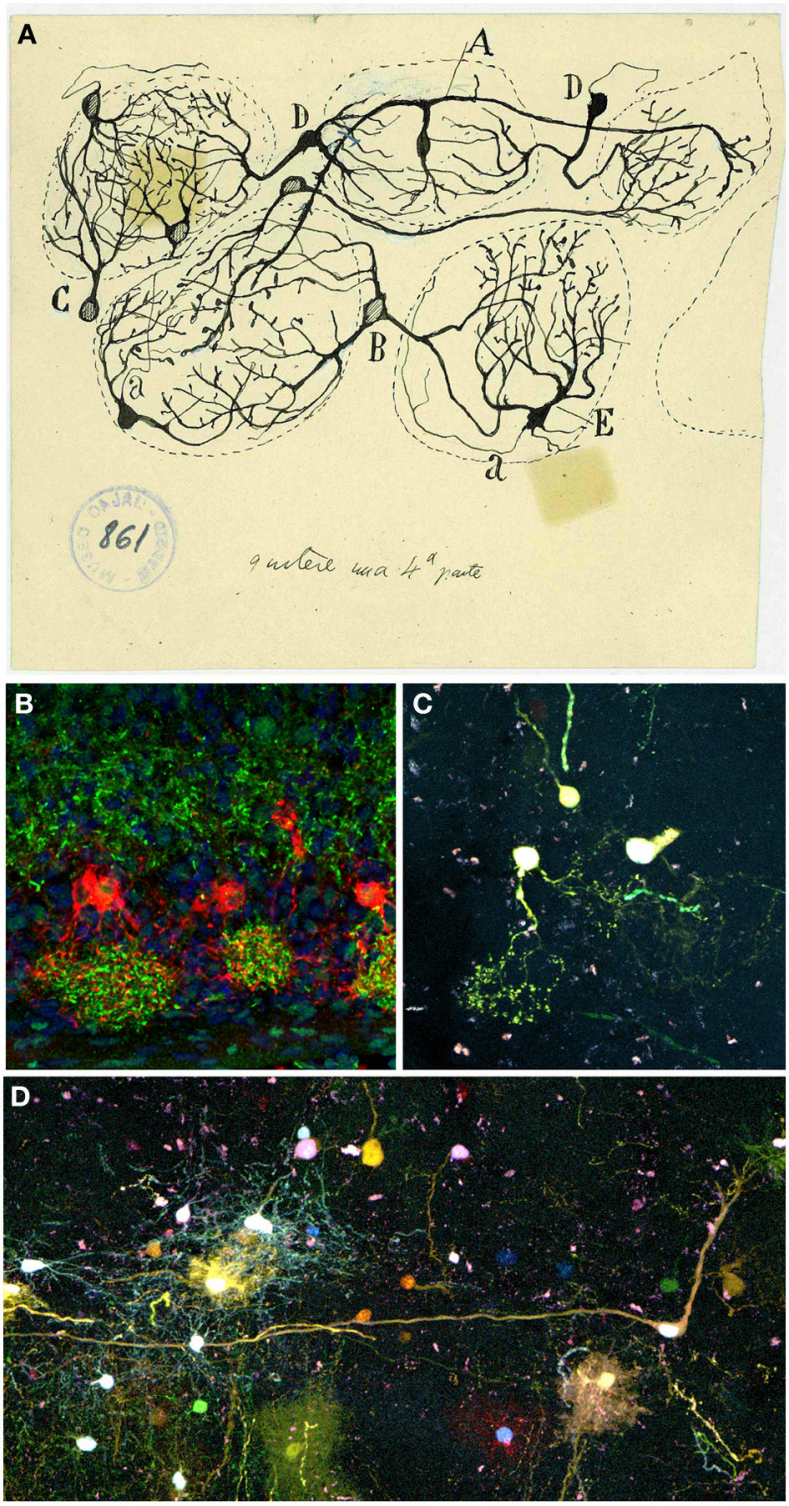
**Periglomerular cells. (A)** Original drawing by Cajal's disciple Blanes, showing periglomerular cells of a cat glomerular layer (Blanes, 1898). Cell branching into three glomeruli *(A)*. Biglomerular cells *(B–D)*. Monoglomerular cells *(C–E)*. *(a)* Axon. Cajal Legacy (Instituto Cajal, CSIC, Madrid, Spain). **(B)** Periglomerular cells immunolabeled for tyrosine hydroxylase (TH, red) protein and Dab1 protein (green) on mouse olfactory bulb sections at P3. Panel was taken by Eduardo Martin-López. **(C,D)** Adult periglomerular cells contributing to one **(C)** or more glomeruli **(D)** labeled after E13 *in utero* electroporation of different plasmids with the *UbC-Star Track method* (Figueres-Oñate and López-Mascaraque, 2013).

Next stratum, the GL, is defined by Cajal as the target of the fibrils coming from the OSNs: “Under the peripheral fibrillar layer lays an irregular area of two or more rows of disordered ovoid masses called olfactory glomeruli. […] They are composed of the terminal branches of olfactory fibers, the thick plume of dendrites arriving from deeper zones, certain tiny nerve corpuscles and, finally, some neuroglial elements” (Ramón y Cajal, 1890b). More than a century ago Cajal stated the exact input of the OSNs into the glomeruli, although Golgi reported the intraglomerular branching of the olfactory fibers (Golgi, 1875). In 1890 Cajal described the composition of each glomerulus (Figure 6A): the terminal arborization of the olfactory fibers, the thick apical dendrites from deeper regions, considerable tiny nervous corpuscles and several neuroglial elements (Ramón y Cajal, 1890b). Those tiny nervous corpuscles correspond to tufted or fusiform nerve cells, that collaborate in the formation of what he called intraglomerular plexus (Figures 6B,C), and external grains or short axon nerve cells, which branch within glomeruli, cells classified by Golgi as glial cells. Nowadays, a further characterization can be achieved either by the specific expression of different markers for each cell type presents or by the cell's physiological properties. Moreover, while Cajal studied the development of this system both in younger and/or phylogenetically less complex animals, nowadays we describe the cellular contributions, e.g., to the OB, after *in utero* viral infections (Blanchart et al., 2011) or by electroporation of different plasmids. In fact, a clonal analysis of glial cell populations can be performed with the Star Track approach (García-Marqués and López-Mascaraque, 2013). Moreover, a modification of this technique that uses an ubiquitous promoter (*UbC-Star Track*, (Figueres-Oñate and López-Mascaraque, 2013) allows a more comprehensive lineage study of all the cell populations (Figure 6D).

Since the apical dendrite of mitral cells and 2–3 dendrites of tufted cells penetrate into the territory of each glomerulus, Cajal noted that “the propagation of the nerve impulse is not individual, from a single neuron to another, but collective, from a group of nerve fibers to a group of ganglion corpuscles” (Ramón y Cajal, 1901). Nowadays, the characterization of the functional glomerular map has led to a more thorough understanding of how the positional domain information translates to different odor responses such as innate or learned responses (reviewed in Mori and Sakano, 2011).

**Figure 7:**
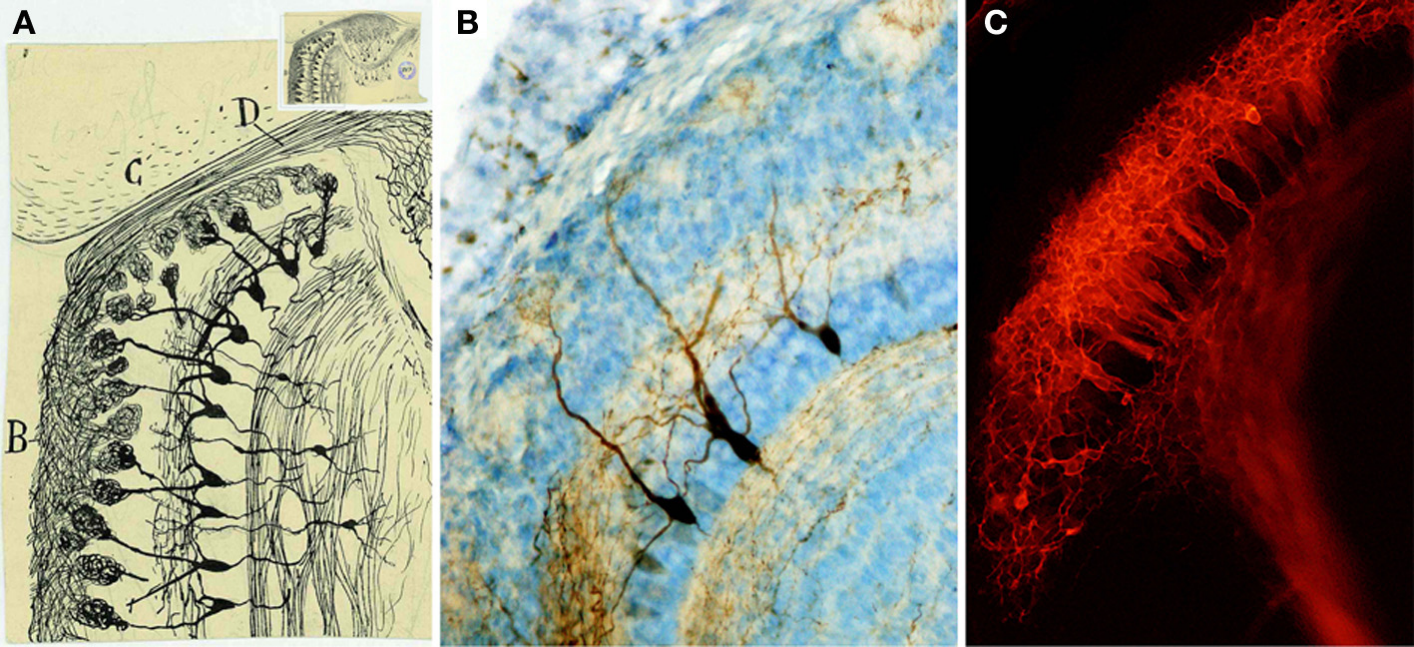
**Mitral cells. (A)** Magnified detail of the original Cajal figure (upper inset). Horizontal mouse olfactory bulb section at 20-days-old (Ramón y Cajal, 1901). Olfactory bulb *(B)*, frontal cortex *(C)*, Olfactory nerve *(D)*. Cajal Legacy (Instituto Cajal, CSIC, Madrid, Spain). **(B)** Mitral cells labeled after BDA injection into the lateral olfactory tract at P5 (Blanchart et al., 2006). **(C)** Retrograde labeling of mitral cells after DiI injection into the lateral olfactory tract at E17 (Blanchart et al., 2006).

Below the glomeruli is located the EPL, similar to the molecular area of the cerebellum or the retina. This layer includes lateral dendrites of the mitral and tufted cells and apical dendritic processes of granular cells (Figure 7A). Cajal named tufted cells as that because of their robust peripheral dendrite branching into the olfactory glomeruli. They are divided into external, middle and deep, dependent on the location of their soma. Cajal also described the presence of axonal collaterals from mitral cells in this molecular layer.

The next stratum is the MCL, composed by mitral cells. Mitral cell bodies, as described by Cajal, form a regular single row and owe their name to their appearance (Figure 7A). They are the principal output cells of the OB and, in most mammals, are characterized by a single apical dendrite through the EPL that branches into an apical tuft within the glomerulus (Figure 7B). Mitral cells are one type of the projection neurons (Figure 7C), whose entire development terminates at postnatal stages (Blanchart et al., 2006). Within the glomeruli, mitral cells interact and receive inputs through synaptic contacts with periglomerular and granule cells. Mitral cells are the bridge connecting directly the periphery with higher integrative structures (Ramón y Cajal, 1904; reviewed in Gire et al., 2013). Their inputs come from OSNs and external tufted cells and send their outputs to various cortical structures (Hayar et al., 2004; Gire et al., 2012). Cajal also described the centrifugal feedback that mitral cells receive from cortical structures (Ramón y Cajal, 1901), and recent studies provided a functional explanation to these projections (for review see Gire et al., 2013).

**Figure 8:**
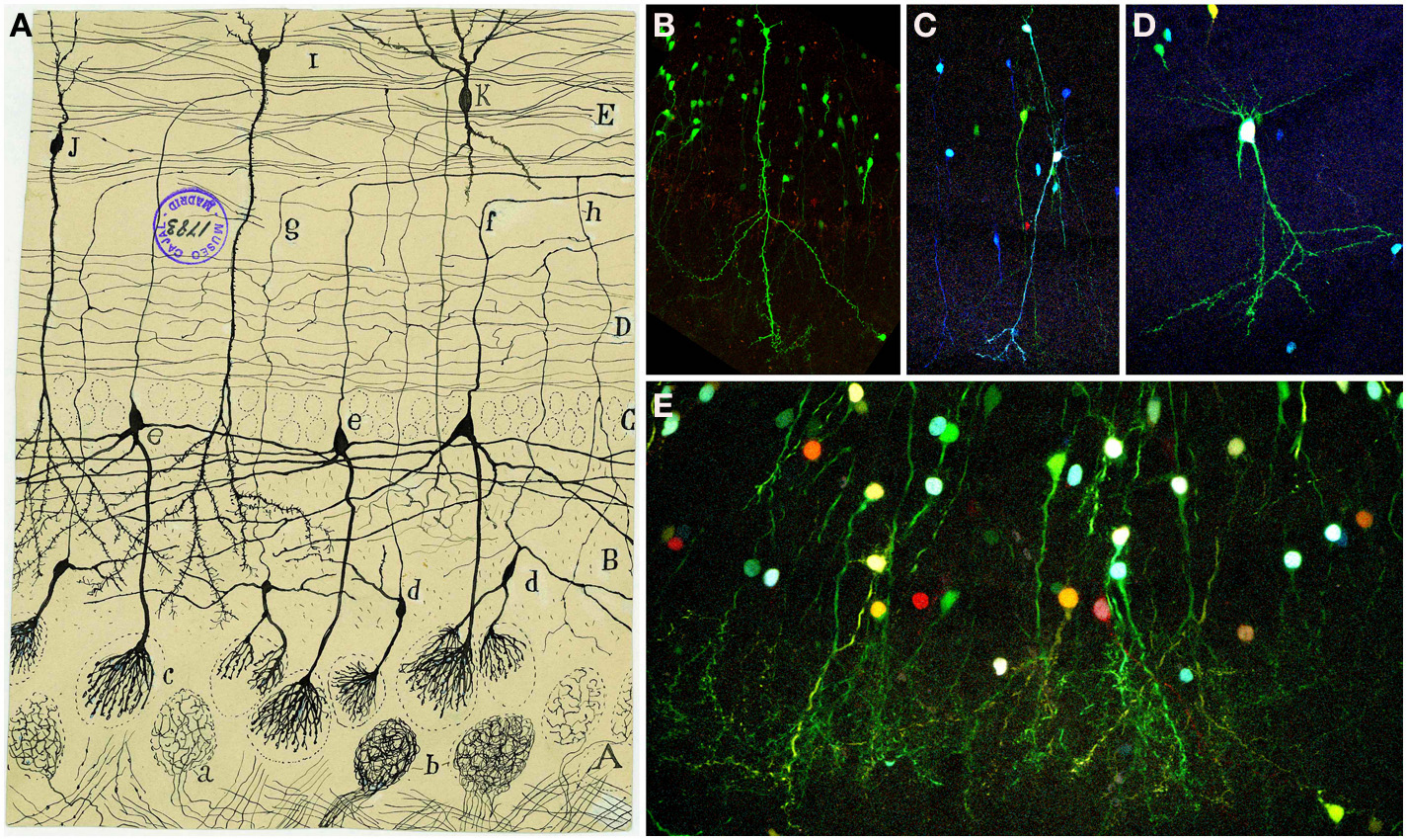
**Granular cells. (A)** Original Cajal drawing of an olfactory bulb section from a few days cat brain (Ramón y Cajal, 1901). *G*lomerular layer *(A)*, outer plexiform layer *(B)*, mitral cell layer *(C)*, inner plexiform layer *(D)*, grains layer and white matter *(E)*. *(a)* Terminal arborization of an olfactory fiber; *(b)* glomerulus with several endings; *(c)* mitral plume; *(d)* tufted cells. Cajal Legacy (Instituto Cajal, CSIC, Madrid, Spain). **(B–E)** Granule cells in the olfactory bulb of young adult mice (P20) labeled after E12-14 *in utero* electroporation of different plasmids with the *UbC-Star Track* method. (Figueres-Oñate and López-Mascaraque, 2013). **(B,C)** Granule cells with similar to (i,j) in Cajal's drawing. **(D)** Tufted cell. **(E)** Granular cells branching their processes in the glomeruli.

Below the MCL layer, the IPL is populated by most axon collaterals of tufted cells (Figure 8A), while the GcL contains many interneurons like the granule cells and the short-axon cells. The granule cells are small spiny ovoid cells with an apical process extending radially into the EPL and short secondary dendrites confined to the GcL (Figures 8B–E). Golgi reported that these cells showed no evidence of axons Golgi (1875) and Blanes (1898) stated that they were not glia as Kölliker claimed (Kölliker, 1891). Additionally, Cajal described different types of short-axon cells within the GcL: Golgi cells, Cajal cells, and Blanes cells. Blanes cells are interneurons with a significant electrophysiological role, as they provide inhibitory inputs onto granule cells and appear to be excited by mitral cells, which could be a novel mechanism for encoding short-term olfactory information (Pressler and Strowbridge, 2006). The different subpopulations of these interneurons were classified by Cajal on morphological and spatial basis. Since his work, the diversity of GcL cells has also been based on molecular and physiological features (Price and Powell, 1970; Schneider and Macrides, 1978; López-Mascaraque et al., 1986; Crespo et al., 2001; Kosaka and Kosaka, 2005).

In summary, Cajal plotted the dynamic scheme of the OB, pointing out the need to give a special significance to the protoplasmic processes of mitral and tufted cells, which penetrate into the glomerulus and are in intimate contact with the olfactory fibrils. The olfactory fibers never depart from the glomerular territory and neither axons of central origin enter in the glomeruli, which is against Golgi's assertion. Cajal was also a pioneer in the description of different axonal projections of tufted and mitral cells to the OC (Ramón y Cajal, 1904). Indeed anatomical and physiological differences suggests that mitral and tufted cells may serve different functions and possibly contribute to different aspects of the olfactory code including perception of odorants (Nagayama et al., 2004; Shepherd et al., 2004). Although mitral and tufted cells innervate different cortical targets, the circuitry and projection sites of the tufted cells are not yet well-understood and are still one of the main focus of research in the field (for review see Mori and Sakano, 2011). Besides, the two main inhibitory interneuron types described by Cajal in the OB have a significant role in the olfactory processing: periglomerular cells mediate lateral inhibition at the level of the glomeruli (Aungst et al., 2003), while granule cells mediate dendrodendritic inhibition onto the lateral collaterals of mitral cells (Schoppa et al., 1998). These synapses formed between lateral dendrites of mitral cells and granule cells was suggested to be inconsistent with Cajal's Law of Dynamic Polarization (for extensive reviews see Shepherd et al., 2007; Sassoè-Pognetto, 2011).

## Neuroglia in the olfactory bulb

Cajal and his colleagues played an important role in describing glial cells. They initiated an active discussion regarding where to encompass those, at that time, unknown cells into the functional map of the brain. In different species, Cajal identified these cells, closely related to the cell bodies of neurons, as neuroglia. Then, he could not draw any definitive conclusion about the physiological role of neuroglial cells, but he presupposed an insulating role: protection to prevent contact between nerve fibers (Ramón y Cajal, 1896). This insulating theory of the neuroglia was originally developed by Cajal's brother, Pedro, and it was always supported by Cajal: “By rational conjecture, we have defended in several manuscripts the thesis, initially suggested by my brother, that both the epithelial and neuroglial cells have a role insulating the fibers and nervous cells, preventing contacts between close but dynamically independent elements” (Ramón y Cajal, 1897).

**Figure 9:**
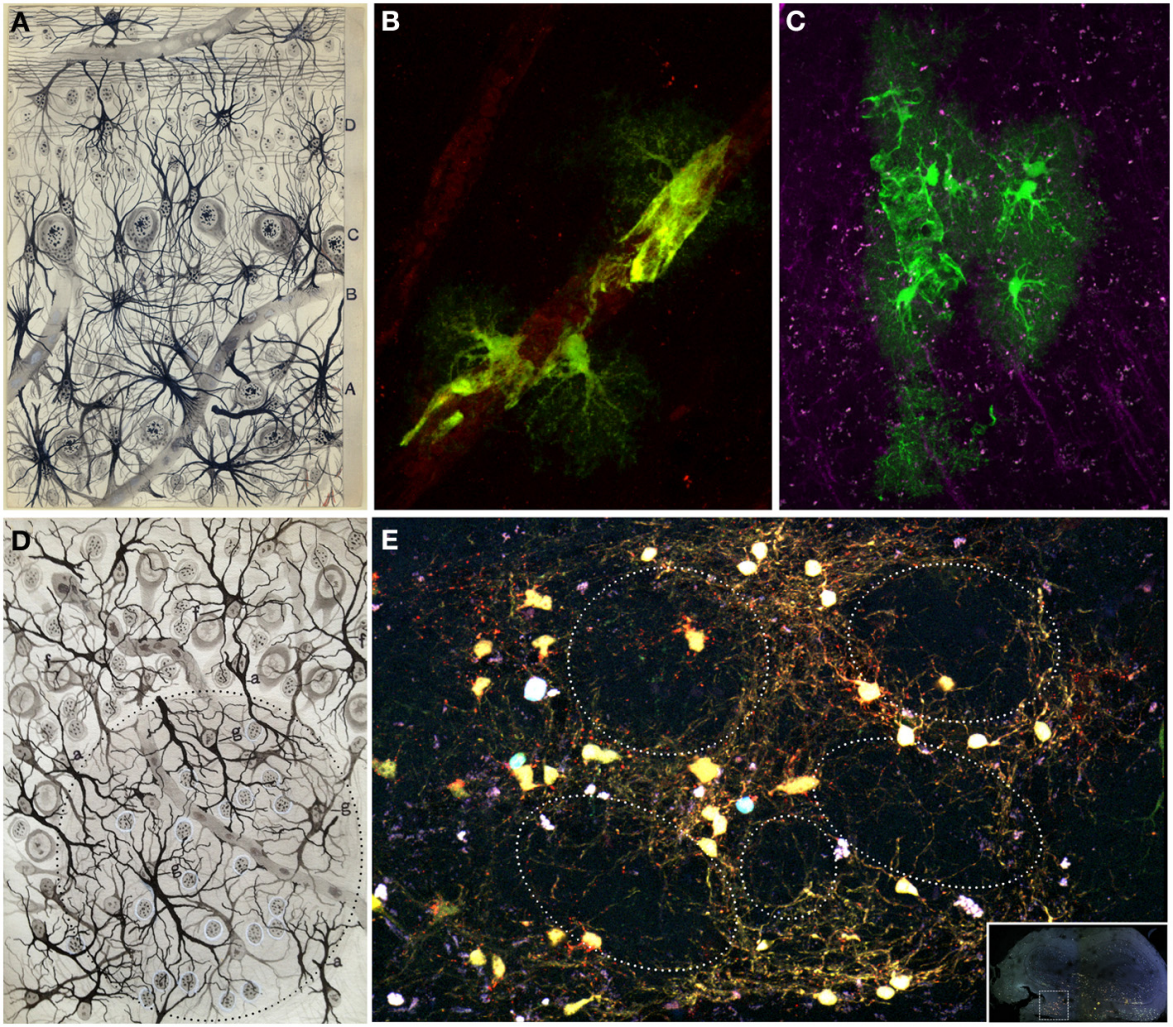
**Glial cells. (A)** Original Fernando de Castro drawing of human olfactory bulb stained with Cajal's gold chloride sublimate method. Superficial substratum of the molecular layer with numerous cephalopodic cells *(A)*, deep substratum *(B)*, mitral cell layer *(C)*, grains layer *(D)* (De Castro, 1920). **(B,C)** Processes of protoplasmic astrocytes arranged around a blood vessel labeled after *in utero* electroporation of the *Star Track* plasmid mix (García-Marqués and López-Mascaraque, 2013) into the lateral ventricles. These perivascular end feet are represented in the olfactory bulb **(A)** by Fernando de Castro. Panel **(B)** modified from Martín-López et al. (2013) and panel **(C)** were taken by Eduardo Martin-López. **(D)** Fernando de Castro drawing illustrating the glomerular layer of the adult dog stained by the Cajal's gold chloride sublimate method. *(g)* Intraglomerular fibrous elements; *(a)* radioglomerular corpuscles; *(f)* fibrous cells located superficial to the molecular zone, displaying most of their extensions oriented toward deepest layers. Note numerous nuclei located in this region, corresponding to Cajal's adendritic glia (De Castro, 1920). **(E)** Clone of glial cells surrounding several glomeruli in an adult (7 months) olfactory bulb labeled after E13 *in utero* electroporation of different plasmids with the *UbC-Star Track* method (Figueres-Oñate and López-Mascaraque, 2013).

Focusing on the OB, Golgi briefly described the glia in this structure, but one the most important descriptions was done by Cajal's disciple, De Castro (1920). De Castro made a careful comparison between the neuroglia of human OB to other higher mammals by using the Cajal-improved sublimated-gold technique and the reduced silver impregnation method (Figures 9A,D). With these staining methods, Fernando de Castro showed the neuroglial distribution in the OB as well as the abundance and importance of the vascular glial end-feet in different brain areas. He also suggested that neuroglial cells may release neuroactive substances and directly participate in neural transmission (De Castro, 1951). In addition, he noticed how astrocytes were closely related to blood vessels through their end-feet, raising questions about their specific function: “What role does the neuroglia play in the vascular foot? Would it be entrusted with any function or would it be just a mere support organ? Difficult in every respect is the solution to the problem” (De Castro, 1920). Recently, the processes of protoplasmic astrocytes arranged around blood vessels (Figures 9B,C) were labeled after *in utero* electroporation of the Star Track plasmid mix (García-Marqués and López-Mascaraque, 2013) into the lateral ventricles. After embryonic electroporation of the OB progenitors with *UbC-Star Track* method (Figueres-Oñate and López-Mascaraque, 2013), clones of glial cells surrounding several glomeruli are located in adult olfactory bulbs (Figure 9E).

While the glomerular structure and neuronal connectivity has been extensively described, both the role and connectivity of neuroglia in the OB have yet to be characterized. Within the OB, astrocytes do not just play an insulating or supporting role, but they are also an active part of the sensory integration in the olfactory glomeruli, interacting with their neuronal counterparts, in a glomerulus-specific manner (Roux et al., 2011). Although the olfactory astroglia was defined as a syncytium, the advent of molecular and genetic techniques changed the experimental approaches to determine the progeny of single cells, shedding light to a further network specialization (Houades et al., 2008). A promising approach is the *in vivo* clonal analysis, Star Track, based on the combinatorial expression of different gene reporters (García-Marqués and López-Mascaraque, 2013; García-Marqués et al., 2014) that makes possible to trace the progeny of targeted GFAP progenitors (Figures 9D,E). Besides the classification based on morphology and location of glial cells, we show the presence homogeneous glial clones which indicates the existence of separate progenitors for each glial population (García-Marqués and López-Mascaraque, 2013).

**Figure 10:**
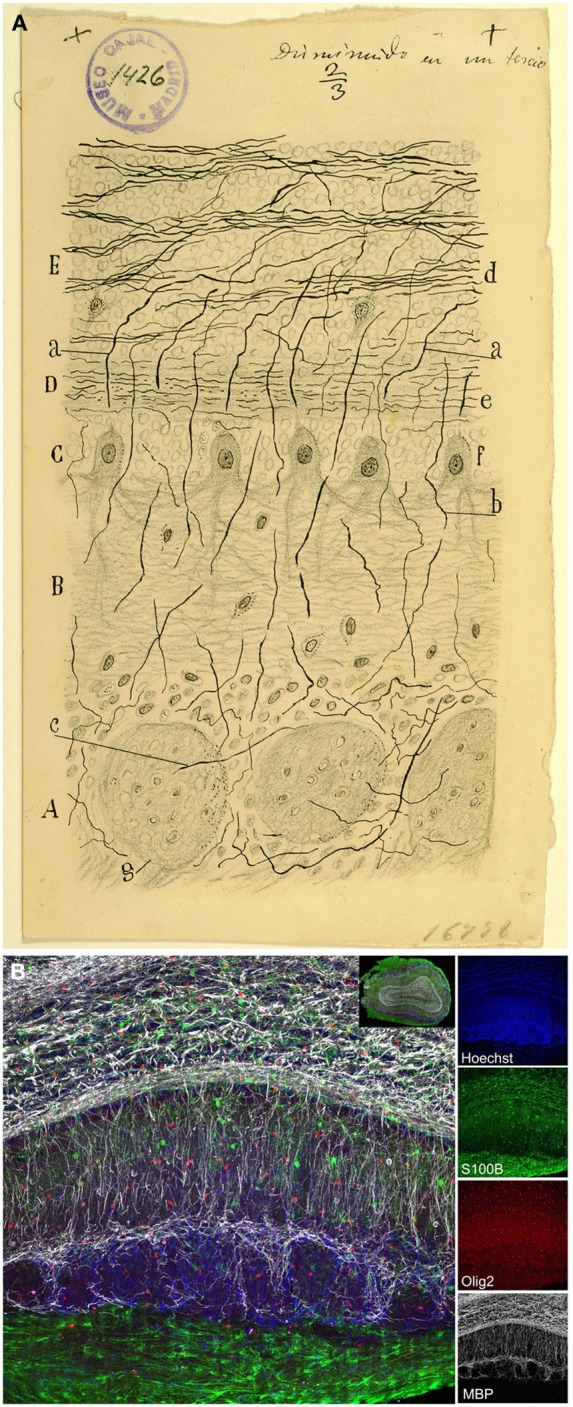
**Myelin. (A)** Original drawing from Cajal of an olfactory bulb section of a month-old rat. Weigert-Pal method (Ramón y Cajal, 1890b). Layer of the glomeruli *(A)*, lower molecular layer *(B)*, mitral layer *(C)*, higher molecular layer *(D)*, coating the grains *(E)*. *(a)* myelin fiber corresponding to the cylinder-axis of mitral cells; *(b)* cylinder-axis of mitral cells; *(c)* core fiber from within the glomeruli; *(d)* bundles of fibers in the layer of grains; *(e)* thin horizontal strands from the higher molecular area; *(f) mitral* cells; *(g)* glomerulus; Cajal Legacy (Instituto Cajal, CSIC, Madrid, Spain). **(B)** Immunohistochemistry with different glial markers: Olig2 (oligodendrocyte progenitors, red), S100β (astrocytes, green), and myelin binding protein (MBP, gray). Nuclei labeled with Hoechst (blue). Inset shows overall view of the olfactory bulb (coronal section) labeled with the markers explained above.

Regardless from the glial elements mentioned above, Cajal also described the presence of myelin fibers within the OB using the Weigert-Pal staining technique (Figure 10A). “The medullated fibers are relatively abundant around the glomeruli and even within them. The periglomerular fibers are generally very thin and correspond with cylinder-axis of the inferior tufted cells […]. The intraglomerular fibers have a more difficult interpretation. […] In general, it can be assumed that said fibers […] end within the same glomerular area. […] As is well known, the olfactory fibers and the grains expansions lack myelin” (Ramón y Cajal, 1890b). Cajal and his disciples also observed what they called “*third element*” or “*adendritic cells*” (De Castro, 1920), known today as microglia. The use of molecular markers, selectively expressed by these cells in the OB, revealed the distinct glia subtypes (Figure 10B).

## Olfactory cortex

The olfactory cortex is a phylogenetically old cortical structure. It is formed by all brain regions receiving direct axonal input from mitral and some tufted cells (Allison, 1954; Price, 1973), making the olfactory system the only sensory modality without thalamic relays. Among several areas, the OC includes the anterior olfactory nucleus, the entorhinal cortex, the piriform cortex (primary OC), *tenia tecta*, cortical amygdaloid nucleus and the olfactory tubercle. Axons from the OB projecting to the OC constitute the lateral olfactory tract (LOT), located on the outer and lower side of the olfactory pedicle named by Calleja and Cajal as the “*external root*” (Calleja, 1893; Ramón y Cajal, 1901).

**Figure 11:**
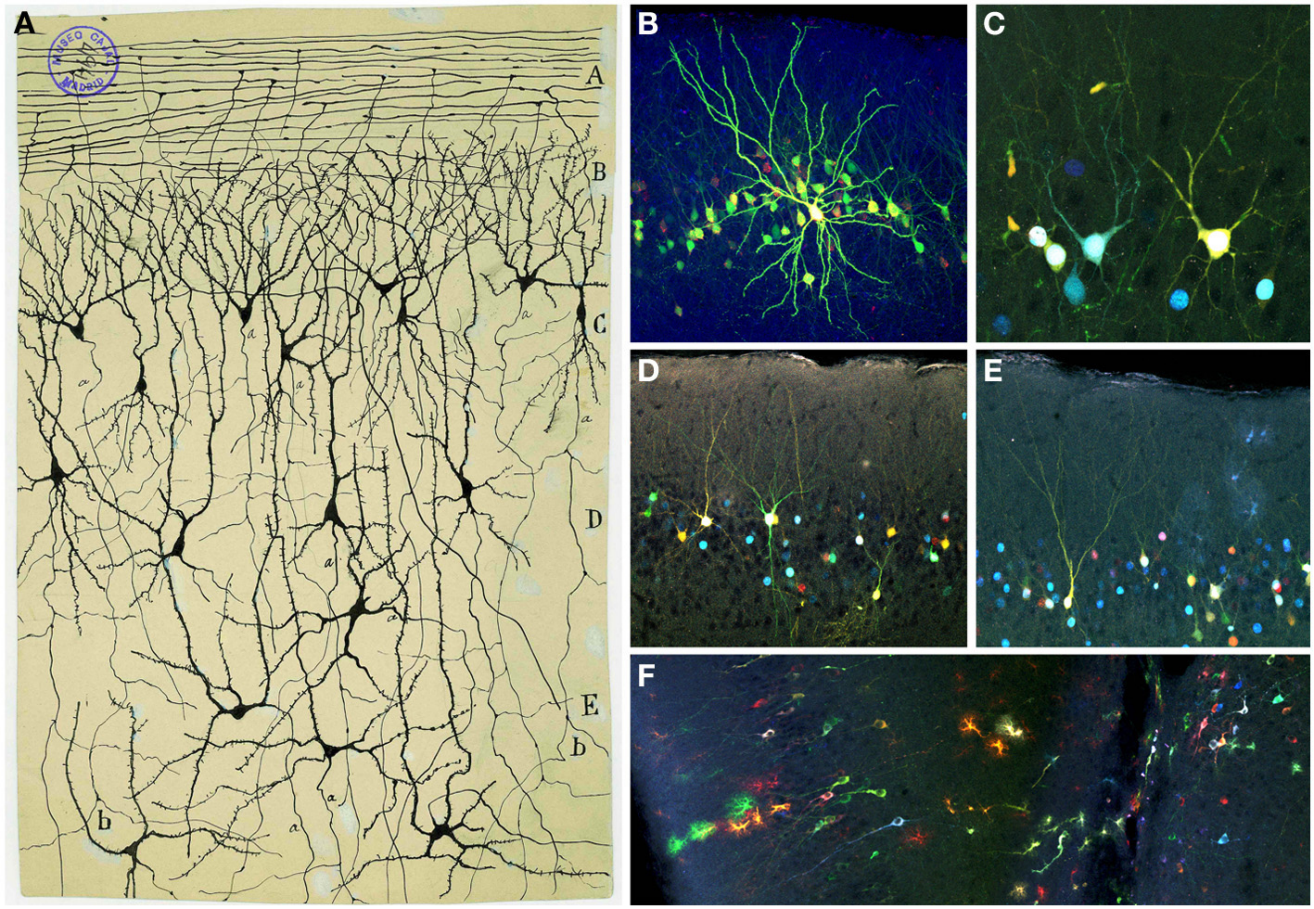
**Olfactory cortex. (A)** Original Cajal drawing showing the olfactory cortex layers (Ramón y Cajal, 1901). *O*lfactory fibers layer *(A)*; plexiform layer *(B)*; layer of polymorphic superficial cells *(C)*; layer of the pyramids *(D);* deep polymorphous cells *(D)*. *(b)* Bifurcation of axons. Cajal Legacy (Instituto Cajal, CSIC, Madrid, Spain). **(B–F)** Different cell morphologies in the adult mouse olfactory cortex labeled after E12 *in utero* electroporation of different plasmids with the *UbC-Star Track method* (Figueres-Oñate and López-Mascaraque, 2013). Note the presence of cells with either arachnoid morphologies, similar to those in **(B)**, and crescent-shaped cells similar to **(C)**. **(D,E)**. View of different morphological neuronal types. **(F)** Several neurons along with different glial clones.

Despite its heterogeneity throughout the rostro-caudal axis, the OC displays a three-layer organization: layer 1 is subdivided in layers 1a and 1b; layer 2 contains semilunar cells and a large number of pyramidal-like cells and layer 3 is formed by different pyramidal cells (Valverde, 1965). Cajal and his disciple Calleja (1893) distinguished five layers in the OC: fibrillar layer or outer root layer, molecular or plexiform layer, layer of small and large pyramids, layer of polymorphs corpuscles and white matter (Figure 11A).

The fibrillar layer (layer 1a) is formed by LOT fibers while the molecular or plexiform layer (layer 1b) receives associational fibers from deeper cells and includes collaterals of the olfactory fibers, tufts of pyramidal cells and dendrites of deeper horizontal cells. The layer of small and large pyramids (layer 2) appears like a “flexible and undulating belt quite well demarcated from the bordering areas” (Ramón y Cajal, 1901). It contains cells with different morphologies, including semilunar cells (superficial part) and a large number of pyramidal-like cells (in deeper regions). While the semilunar cells usually lack descendent axonal projections, deeper cells display axonal processes penetrating into the white matter. As Cajal postulated, “the configuration of the neurons from said layer is highly variable, being able to discover, even in the deepest planes, multiple elements whose shape is triangular, stellated or fusiform, though they never lack a radial dendrite directed to the second layer” (Ramón y Cajal, 1901). At the deepest level, the polymorph cells layer (layer 3) includes the most voluminous cells with descending axonal collaterals that penetrate into the white matter. Recently, the development of novel tools for the clonal analysis of the brain neural lineages, the *UbC-Star Track* method (Figueres-Oñate and López-Mascaraque, 2013), evidenced the large variety of morphologies within the OC (Figures 11B–F). Finally, the white matter is formed by the concurrence of axonal projections from overlying layers, forming a labyrinthic and irregular plexus, that complicates the definition of their routes: “In summary, the fibers or second order conductors coming from the bulbar and frontal cortex, underlying the external root, follow two routes: ones, the majority, go backwards deeply to reach the corpus striatum incorporating to the corona radiata; others travel toward the inside and backwards and enter the anterior commissure. Being unable to sufficiently follow those conductors, we ignore if any of them reach Ammon's horn” (Ramón y Cajal, 1901).

This anatomical organization may underlie the fact that mitral and tufted cells project to the OC through different pathways and toward different targets suggesting the possibility that they carry different odor information (reviewed in Mori and Sakano, 2011). The diverse cortical projections of a single mitral cell, the broad distribution of mitral cells axons and the overlapping of their information at their target neurons provide the basis for a diversification and combinatorial integration of the olfactory information processing (Ghosh et al., 2011). Recent work using anatomical and physiological techniques demonstrated that individual neurons in the piriform cortex receive convergent input from mitral/tufted cells connected to multiple glomeruli located all over the OB (Apicella et al., 2010; Davison and Ehlers, 2011; Miyamichi et al., 2011). The precise scheme of the olfactory pathway displayed by Cajal (Figure 1A) opened the door to the anatomical basis of olfactory processing (Gire et al., 2013). OSNs expressing the same odorant receptor converge in one glomeruli of each hemisphere (Mombaerts et al., 1996; Mombaerts, 2006). This spatial pattern, termed *odotopic map* is just applied for the first olfactory station (OSN to OB). However, although much is known about how odors are represented at the level of OB, the nature of odor representations in this cortex and the integration of the odor activity of output OB neurons into higher brain regions, essential for the cortical odor representations, are still debated (for review see Bekkers and Suzuki, 2013).

## Olfactory system perspectives

“The functional specialization of the brain imposes to the neurons two main gaps: inability to proliferate and irreversibility of the intra-protoplasmatic differentiation. It is because this reason that, once development is over, the growth and regeneration of axons and dendrites are irrevocably dried up. In the adult brains the nervous pathways are fixed, finished, immutable. Everything may die nothing is regenerated itself. It belongs to the science of the future to change, if possible, this cruel decree” (Ramón y Cajal, 1913).

Unlike other brain structures, the OB is not a simple relay nucleus, but a center for information processing and storage. Cajal missed one of the most important characteristics of the OB, the cell turnover: “Nature has given us a limited amount of brain cells. Here is a capital, large or small, that nobody can increase as the neuron is unable to multiply” (Ramón y Cajal, 1931). However, adult neurogenesis is among the most important brain discoveries opening new debate about the function and integration of these cells into the system. The adult mice brain retains a proliferative area, the subventricular zone (SVZ), which maintains proliferative functions through live. Astrocyte-like cells (B cells) divide to produce neuroblasts via intermediate progenitors. These neuroblasts migrate along the rostral migratory stream to the OB, where they differentiate and migrate to their final positions in the granular or periglomerular layers (Kriegstein and Alvarez-Buylla, 2009). Strikingly, a spatial patterning within the SVZ indicates that interneuron subtypes depend on their generation area (Merkle et al., 2007). Moreover, the temporal differences in the production of interneurons are related to their subtype specification and functional integration in the system (Batista-Brito et al., 2008).

To conclude, Cajal opened up an essential work to our current understanding of this system. These classical studies provided the basis for anatomical, physiological, and molecular studies. Now, more than a century later, the use of state-of-the-art approaches such as cell type specific optogenetic manipulations, *in utero* electroporation, *in vivo* genetic fate mapping and cell ablation, electrophysiological and live-cell imaging techniques, patch-clamp recordings and two-photon microscopy *in vivo* and in brain slice preparations can help understanding how odor information is represented and processed by the olfactory system.

### Conflict of interest statement

The authors declare that the research was conducted in the absence of any commercial or financial relationships that could be construed as a potential conflict of interest.
